# Suspected Pancreatic-Pleural Fistula Presenting With Massive Pleural Effusion Complicated by Fatal Re-expansion Pulmonary Edema

**DOI:** 10.7759/cureus.106393

**Published:** 2026-04-03

**Authors:** Ashwin Sidhu

**Affiliations:** 1 Department of Internal Medicine, Santa Clara Valley Medical Center, San Jose, USA

**Keywords:** pancreatic-pleural fistula, pancreatitis, pleural effusion, re-expansion pulmonary edema, respiratory failure

## Abstract

Pancreatic-pleural fistula (PPF) and re-expansion pulmonary edema (RPE) are rare but potentially fatal complications of pancreatitis. This report describes a 31-year-old man with a history of recurrent alcohol-associated pancreatitis who presented with acute hypoxic respiratory failure due to a large pleural effusion. Chest tube drainage revealed amylase-rich fluid, highly suggestive of a PPF. Rapid drainage of a large volume of pleural fluid precipitated RPE, followed by rapid clinical deterioration and cardiac arrest. This case highlights the importance of recognizing suspected PPF as a cause of large pleural effusions and the need for cautious drainage to prevent life-threatening complications such as RPE. To date, the coexistence of PPF complicated by fatal RPE has rarely been reported.

## Introduction

Pancreatitis is primarily an intra-abdominal inflammatory condition, but it can rarely result in extra-abdominal complications involving the thoracic cavity, particularly in patients with recurrent pancreatitis. Pancreatic-pleural fistula (PPF) is an uncommon sequela of recurrent pancreatitis caused by the disruption of the pancreatic duct, allowing enzyme-rich fluid to track through diaphragmatic hiatuses into the pleural space, where it accumulates and forms large effusions. Pleural effusions due to PPF are extremely rare, accounting for less than 1% of all pleural effusions [1]. These effusions arise from fistulous communication between the pancreas and pleural cavity and are often misdiagnosed due to predominant respiratory symptoms [1]. Diagnosis typically requires pleural fluid analysis, demonstrating markedly elevated pleural fluid amylase levels [1,2].

Management of these effusions is challenging, as drainage alone does not address the underlying pancreatic pathology and carries significant risk. Rapid removal of large volumes of pleural fluid can precipitate re-expansion pulmonary edema (RPE), a rare but potentially fatal complication characterized by acute hypoxia, respiratory distress, and hemodynamic instability. RPE is a condition that occurs following rapid evacuation of large amounts of air or fluid from the pleural space, even in the absence of complete lung collapse [3]. While the exact mechanism is not fully understood, RPE is associated with increased pulmonary capillary permeability and inflammation-mediated injury [3]. Additional mechanisms include surfactant depletion, reactive oxygen species generation during reperfusion, and cytokine-mediated alveolar flooding [3,4].

This case highlights the coexistence of suspected PPF and RPE, emphasizing the importance of early recognition, cautious pleural drainage, and multidisciplinary management to prevent catastrophic outcomes.

This article was previously presented as a meeting abstract at the SHM Sacramento Chapter Scientific Abstract Competition held in Sacramento, California, on November 1, 2025.

## Case presentation

A 31-year-old man with a history of recurrent alcohol-associated pancreatitis presented to the emergency department with four days of progressively worsening shortness of breath. His medical history was notable for approximately five prior episodes of pancreatitis, with the most recent episode occurring five months prior to presentation. His social history was notable for ongoing alcohol use, daily marijuana use, no tobacco use, recent relocation to the area, and employment in information technology. He had a BMI of 27 kg/m^2^.

On arrival, he was in moderate respiratory distress with a heart rate of 135 beats per minute, a respiratory rate of 25 breaths per minute, and an oxygen saturation of 90% on room air. His blood pressure and temperature were within normal limits.

Physical examination revealed increased work of breathing with accessory muscle use and absent breath sounds over the right hemithorax. There was no tracheal deviation. Cardiovascular examination demonstrated tachycardia with a regular rhythm and no murmurs. There was no jugular venous distention, peripheral edema, or other signs of fluid overload. Abdominal examination was benign, with a soft, non-distended, non-tender abdomen. Bowel sounds were present.

Initial laboratory studies revealed leukocytosis, mild anemia, and a markedly elevated serum lipase level. Basic metabolic panel, troponin, NT-proBNP, and liver function tests were otherwise unremarkable. Laboratory evaluation included triglycerides and IgG4, both within normal limits to rule out other causes of pancreatitis (Table 1).

**Table 1:** Initial laboratory studies ALT: alanine aminotransferase; AST: aspartate aminotransferase; ALP: alkaline phosphatase; LDH: lactate dehydrogenase.

Test	Value	Normal
Hemoglobin	11.4 (low)	13.5-17.5 g/dL
White blood cells	14,330 (high)	3,900-10,600 cells/mcL
Sodium	135	135-147 mmol/L
Potassium	3.8	3.5-5.0 mmol/L
Chloride	98	96-106 mmol/L
Blood urea nitrogen	8	5-25 mg/dL
Creatinine	0.8	0.7-1.4 mg/dL
Glucose	103	70-200 mg/dL
Lipase	2,092 (high)	13-63 U/L
Triglycerides	104	0-150 mg/dL
IgG4	98	1-123 mg/dL
Troponin	11	<22 ng/L
NT-proBNP	104	<125 pg/mL
Total bilirubin	0.4	0.2-1.0 mg/dL
Total protein	5.8 (low)	6.0-8.0 g/dL
Alanine aminotransferase (ALT)	8	0-40 U/L
Aspartate aminotransferase (AST)	24	0-37 U/L
Alkaline phosphatase (ALP)	87	40-129 U/L
Lactate dehydrogenase (LDH)	323 (high)	118-242 U/L

**Figure 1:**
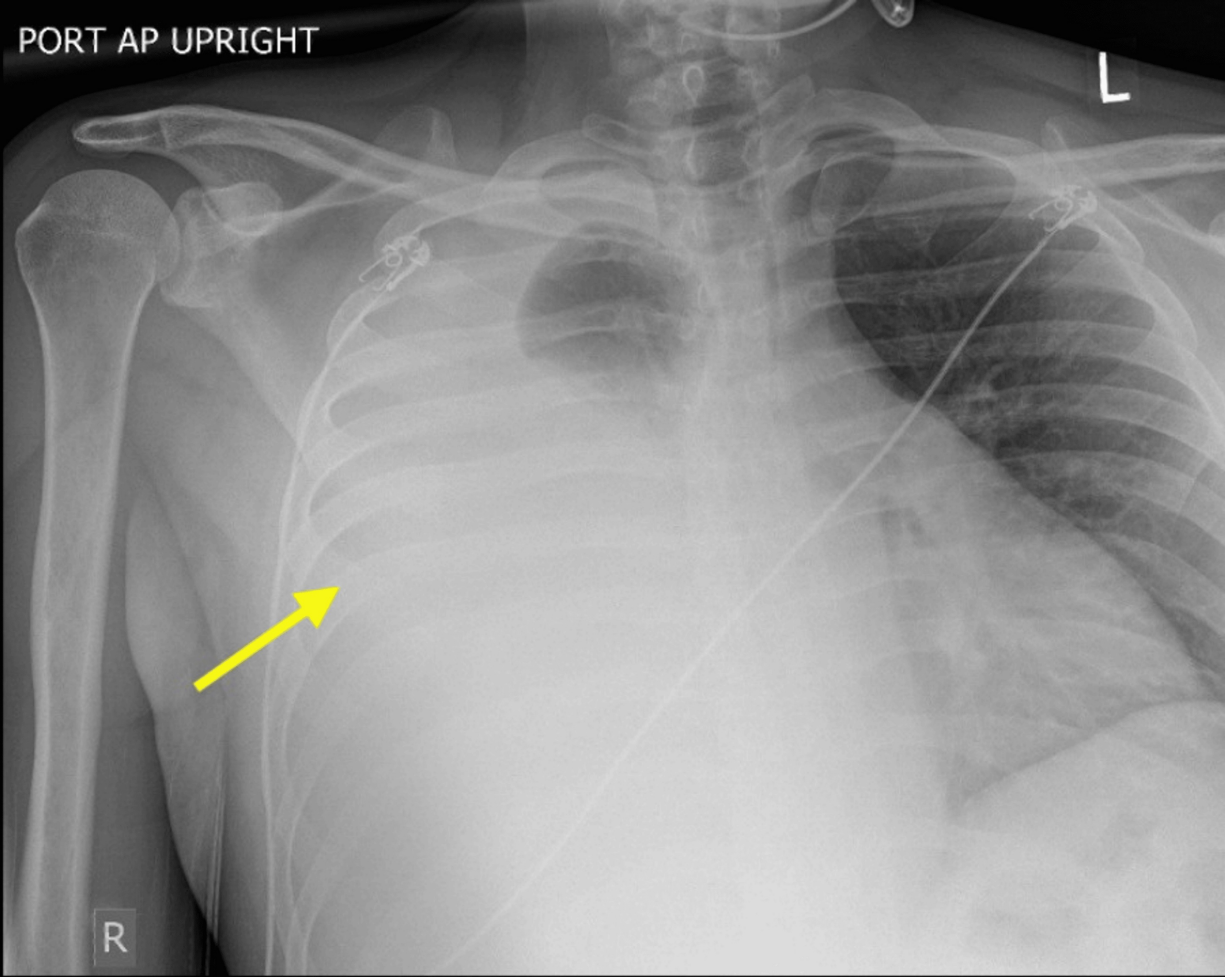
Initial chest X-ray The initial chest X-ray demonstrated opacification of the right hemithorax with minimal aeration of the right upper lung, reflecting a large pleural effusion (yellow arrow).

Chest imaging demonstrated a large, right-sided pleural effusion with significant associated lung collapse (Figure 1).

Computed tomography (CT) imaging was not performed because the patient was too unstable from a respiratory standpoint to safely undergo transport and imaging.

**Figure 2:**
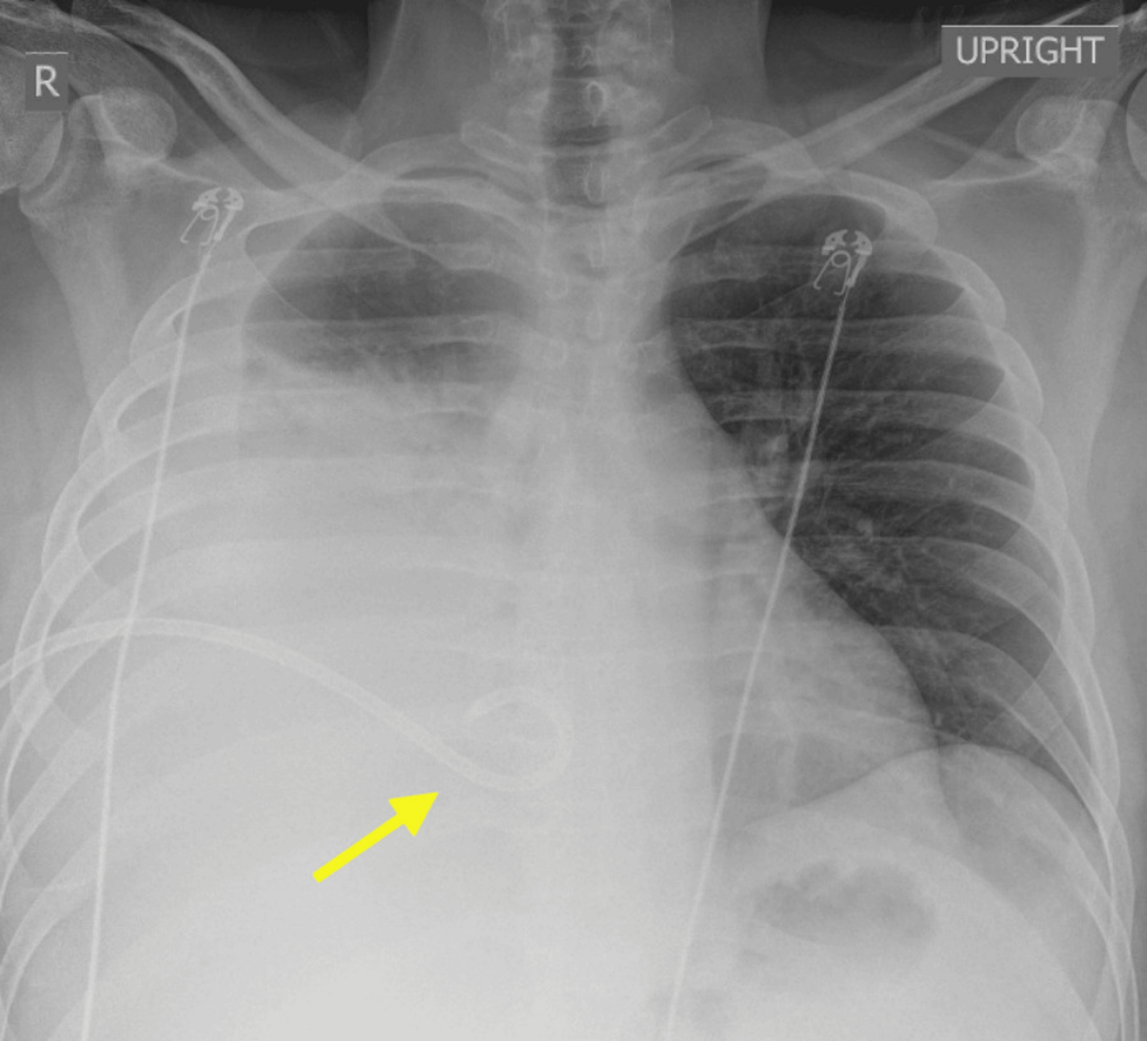
Chest X-ray after pigtail chest tube placement Pigtail chest tube projected over the right lower medial chest (yellow arrow) with a decrease in the pleural effusion noted in the initial chest X-ray.

Given the extent of the effusion and degree of lung collapse, a chest tube was placed for both diagnostic and therapeutic purposes. Upon insertion, approximately 1.5 liters of exudative pleural fluid were immediately evacuated under water seal. The chest tube was subsequently clamped. Repeat chest imaging noted a decrease in the pleural effusion (Figure 2).

Pleural fluid analysis revealed markedly elevated amylase levels, raising strong suspicion for a pancreatic origin of the effusion (Table 2).

**Table 2:** Pleural fluid studies Pleural fluid studies were consistent with an exudative effusion per Light’s criteria (protein ratio: 0.60, LDH ratio: 2.58). The markedly elevated pleural fluid amylase level was highly suggestive of a pancreatic-pleural fistula. LDH: lactate dehydrogenase.

	Pleural fluid	Normal (pleural fluid)
Total protein	3.5	<3.0 g/dL
Lactate dehydrogenase (LDH)	835	<200 U/L
Amylase	20,900	<150 U/L
Glucose	81	60-100 mg/dL
Triglycerides	68	<50 mg/dL (non-chylous), >110 mg/dL (chylous)
White blood cells	1,454	<1,000 cells/µL
Red blood cells	36,000	Minimal/near 0
Total nucleated cells	1,462	<1,000 cells/µL
Differential	Neutrophils 6.5%, lymphocytes 3.0%, mononuclear 76.5%, eosinophils 14%	Variable
Fluid gross appearance/color	Markedly bloody	Clear, straw-colored
Gram stain	No organisms	No organisms
Culture	No growth	No growth
Cytology	Negative	Negative
pH	Not obtained	7.60-7.66
Amylase isoenzyme	Not obtained	-

Further diagnostic evaluation with magnetic resonance cholangiopancreatography (MRCP) was planned to evaluate for a suspected PPF. Gastroenterology was consulted, and octreotide therapy was empirically initiated, with consideration for endoscopic pancreatic duct stenting pending imaging results.

Approximately 14 hours later, after transfer to the medical ward, the chest tube was returned to water seal given concern for persistent large-volume effusion and incomplete lung re-expansion. An additional 1.3 liters of pleural fluid drained over approximately 20 minutes.

**Figure 3:**
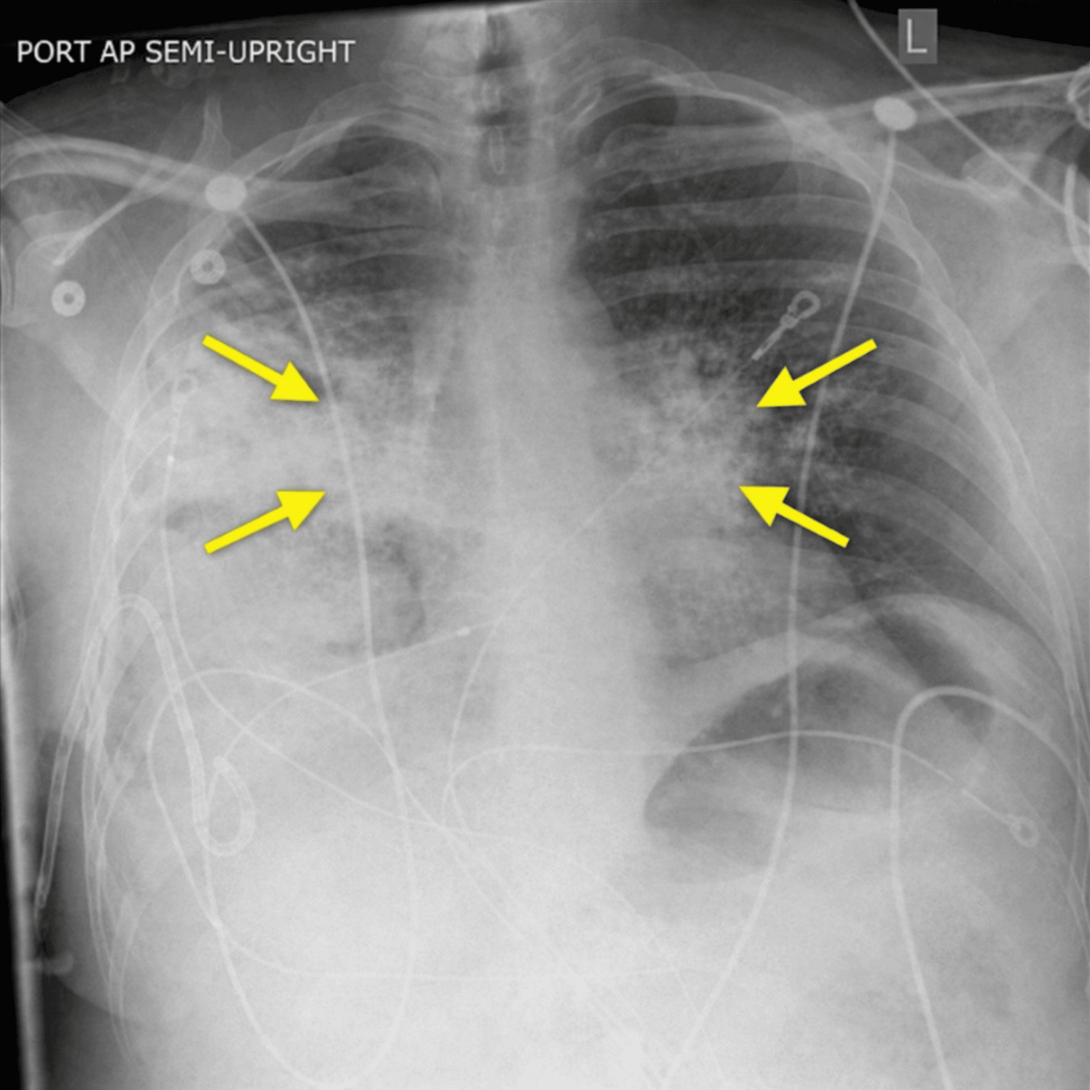
Chest X-ray consistent with re-expansion pulmonary edema Chest imaging demonstrating bilateral perihilar airspace opacities consistent with pulmonary edema, with yellow arrows highlighting areas of alveolar opacification.

Shortly after the second drainage, the patient developed acute worsening respiratory distress characterized by increased work of breathing and production of pink, frothy sputum. His oxygen requirements escalated rapidly, and clinical findings were concerning for RPE. Repeat chest imaging demonstrated new bilateral perihilar pulmonary opacities (Figure 3).

The patient was transitioned to high-flow nasal cannula and transferred to the medical intensive care unit for close monitoring and advanced respiratory support. Despite supportive measures, his condition deteriorated rapidly, progressing to hemodynamic instability and impending shock. He required endotracheal intubation for airway protection and respiratory failure. Shortly thereafter, he developed pulseless electrical activity (PEA) arrest. Advanced cardiac life support protocols were initiated, including administration of multiple vasopressors and resuscitative interventions; however, return of spontaneous circulation was not achieved, and the patient was pronounced deceased.

A timeline of events is summarized in Table 3.

**Table 3:** Timeline of clinical events PEA: pulseless electrical activity; ED: emergency department.

Sequence	Event	Timing	Details/Notes
1	Chest tube placement	About two hours after the presentation	Performed in ED for a large right pleural effusion
2	Initial drainage (1.5 liters)	Shortly after chest tube placement	Rapidly evacuated under water seal; exact drainage rate not documented
3	Chest tube clamped	Immediately after initial drainage	No predefined staged drainage protocol
4	Duration of clamping	~14 hours	Patient remained clinically stable during this interval
5	Second drainage (1.3 liters)	After ~14 hours, upon return to the water seal	Approximately 1.3 liters drained over ~20 minutes
6	Symptom onset	Within ~20 minutes of second drainage	Cough, hypoxia, oxygen desaturation, and hypotension
7	ICU transfer	Immediately after decompensation	Rapid escalation of care
8	Follow-up chest imaging	Shortly after decompensation	Demonstrated new bilateral pulmonary edema
9	Cardiac arrest (PEA)	Approximately two hours after ICU admission	Patient was already intubated prior to arrest

## Discussion

PPF is typically caused by disruption of the pancreatic duct or rupture of a pseudocyst, allowing pancreatic secretions to track into the pleural space [1,2,5]. Diagnosis relies on a high index of suspicion, particularly in patients with recurrent pancreatitis or unexplained pleural effusions. Pleural fluid analysis demonstrating markedly elevated amylase is highly suggestive, and imaging with MRCP is essential for confirming the fistula and identifying the site of ductal disruption [1,3,5].

Pleural fluid amylase levels exceeding 1,000 U/L have been reported to be highly suggestive of pancreatic origin [6]. In this case, the pleural fluid amylase exceeded 20,000 U/L, strongly supporting a pancreatic source. Additional pleural fluid studies (Table 2) demonstrated an exudative effusion with negative cultures and cytology, making infection and malignancy less likely. Although triglyceride levels were in a borderline range, chylothorax was considered less likely given the markedly elevated amylase level.

Despite these findings, confirmatory imaging with MRCP was planned but not completed due to rapid clinical deterioration. Additionally, amylase isoenzyme testing was not performed. As such, the diagnosis remains a suspected PPF, representing an important limitation of this case.

PPF typically presents with respiratory symptoms such as dyspnea, cough, and chest pain, with only about one quarter of patients reporting abdominal pain [1]. Consistent with this, the patient in this case did not report abdominal pain on presentation.

Recent case reports emphasize that early recognition and targeted endoscopic management, such as pancreatic duct stenting, can prevent recurrence and reduce morbidity [7,8]. Management of PPF requires more than symptomatic pleural drainage. Conservative measures, including bowel rest and somatostatin analogs such as octreotide, may be appropriate for stable patients with smaller effusions [1,2,5]. In this case, octreotide therapy was initiated; however, MRCP was not completed due to rapid clinical deterioration, and the diagnosis remained a suspected PPF. Additionally, cross-sectional abdominal imaging was not obtained due to hemodynamic instability, limiting evaluation for pancreatic duct disruption, pseudocyst formation, or chronic pancreatitis.

RPE is a rare but life-threatening complication following rapid evacuation of large pleural effusions. Its incidence is less than 1%, but reported mortality approaches 20% [9]. Mechanisms include increased pulmonary capillary permeability, reperfusion injury, and mechanical stress during rapid lung re-expansion [3,9]. Clinically, it manifests within hours as acute hypoxemia, respiratory distress, and pink frothy sputum. Literature indicates that removing volumes exceeding 1-1.5 liters at once significantly increases the risk [3,9].

As outlined in Table 3, the patient developed acute respiratory decompensation within 20 minutes of this second drainage event, strongly supporting a temporal association with RPE. The initial rapid evacuation may have contributed to “priming” of the pulmonary vasculature, with subsequent drainage precipitating capillary leak and pulmonary edema.

Notably, imaging demonstrated bilateral pulmonary edema despite unilateral drainage, a phenomenon that has been described in the literature and is thought to result from systemic inflammatory responses, pulmonary vascular redistribution, and increased capillary permeability [10,11]. This supports the concept that RPE is not solely a localized mechanical process but may involve systemic pathophysiologic mechanisms [10,11].

Although RPE may be self-limited in mild cases, severe presentations can progress rapidly and carry significant mortality, as demonstrated in this case. This emphasizes the importance of early recognition and close monitoring [9,12]. Management is primarily supportive and requires early recognition and rapid escalation of care. Initial treatment includes supplemental oxygen, with progression to non-invasive ventilation or invasive mechanical ventilation with positive end-expiratory pressure (PEEP) in severe cases [13]. Hemodynamic instability may require vasopressor support and fluid resuscitation, as clinically indicated. Any ongoing negative pressure drainage should be discontinued immediately. Additional strategies described in the literature include lateral decubitus positioning with the affected side up in unilateral cases. The role of pharmacologic therapies, including diuretics or anti-inflammatory agents, remains variable and should be guided by clinical judgment [13].

In this case, alternative causes of acute decompensation were considered. Cardiogenic pulmonary edema was less likely given normal troponin and NT-proBNP levels and absence of clinical signs of volume overload; however, echocardiography was not obtained prior to deterioration, limiting full assessment. Pulmonary embolism could not be definitively excluded, although empiric thrombolysis was administered during cardiac arrest. Tension physiology post-chest tube was not consistent with chest imaging. Acute respiratory distress syndrome, aspiration pneumonitis, and sepsis were also considered but were less consistent with the abrupt onset immediately following rapid pleural drainage. Thus, while definitive exclusion of all alternative etiologies was limited, the temporal relationship and clinical presentation strongly support RPE as the primary cause of deterioration.

Overall, the coexistence of PPF and RPE represents a convergence of two distinct pathophysiologic processes. In PPF, prolonged accumulation of enzyme-rich fluid leads to sustained lung compression and reduced ventilation, often over days to weeks. This chronic collapse predisposes the pulmonary microvasculature to injury upon rapid re-expansion.

This case, in conjunction with the existing literature, emphasizes three key points: (1) PPF should be suspected in patients with large or recurrent pleural effusions and a history of pancreatitis; (2) pleural fluid analysis and advanced imaging are crucial for timely diagnosis; and (3) rapid large-volume drainage carries a significant risk of RPE, particularly when the underlying fistula cannot be immediately managed, necessitating staged decompression, supportive care, and close monitoring.

## Conclusions

PPF is a rare but important cause of large pleural effusions, particularly in patients with a history of pancreatitis presenting with respiratory symptoms. This case underscores the diagnostic importance of pleural fluid analysis, especially markedly elevated amylase levels, in identifying this condition. It also highlights the potentially catastrophic risk of RPE following rapid, large-volume pleural drainage. Careful, staged drainage and close clinical monitoring are essential to reduce this risk. Early multidisciplinary management addressing the underlying pancreatic pathology is critical to improving patient outcomes.
